# Psycho-oncology service provisions for hospitalised cancer patients before and during the COVID-19 pandemic in an oncology centre in eastern India

**DOI:** 10.3332/ecancer.2021.1226

**Published:** 2021-05-10

**Authors:** Arnab Mukherjee, Meheli Chatterjee, Shreshta Chattopadhyay, Chitralekha Bhowmick, Archisman Basu, Surya Bhattacharjee, Soumita Ghose, Soumitra Shankar Datta

**Affiliations:** 1 Department of Palliative Care & Psycho-oncology, Tata Medical Centre, New Town, Rajarhat, Kolkata, West Bengal 700160, India; 2 Department of Medical Oncology, Tata Medical Centre, New Town, Rajarhat, Kolkata, West Bengal 700160, India; 3 Department of Quality Assurance, Tata Medical Centre, New Town, Rajarhat, Kolkata, West Bengal 700160, India; 4 Department of Medical Administration, Tata Medical Centre, New Town, Rajarhat, Kolkata, West Bengal 700160, India; 5 MRC Clinical Trials Unit, Institute of Clinical Trials and Methodology, University College London, Gower St, London WC1E 6BT, UK; a https://orcid.org/0000-0002-6325-7116; b https://orcid.org/0000-0001-6964-0282; c https://orcid.org/0000-0003-4363-0910; d https://orcid.org/0000-0002-6325-7116; e https://orcid.org/0000-0001-9958-3709; f https://orcid.org/0000-0003-0084-1283; g https://orcid.org/0000-0003-1674-5093

**Keywords:** SARS-CoV-2, COVID-19, cancer, oncology, psychological support, psychiatry, psycho-oncology

## Abstract

**Background:**

Addressing the mental health needs of cancer patients and their caregivers improves the quality of care the patient receives in any cancer care ecosystem. International practice currently encourages integrated care for physical and mental health in oncology. The coronavirus disease (COVID-19) pandemic has affected the delivery of healthcare services across the world. The current research paper is on the psycho-oncology service provision for hospitalised cancer patients before and during the COVID-19 pandemic.

**Methods:**

All patients who were referred to psycho-oncology services during the study period of 1 month, in the two successive years of 2019 and 2020, were included in the study. Retrospective data were collected from the centralised electronic medical records for patients. Data included cancer diagnosis, reason for admission, admitting team and reason for a psychiatric referral. Other parameters that were measured were the timing of the psychiatric assessment, psychiatric diagnosis and psycho-oncology care provided, which included psychological interventions carried out and medications prescribed. The overall institutional data on cancer care provision are also presented in brief to provide context to the psycho-oncology services.

**Results:**

Integrated psycho-oncology services reviewed and managed patients round the year in the hospital where the study was conducted. During the 1-month study period, in 2019 and 2020, the total number of hospitalised cancer patients managed by the services was 74 and 52, respectively. During the study period of 2020, 292 patients with cancer who were being treated in the hospital had tested positive for severe acute respiratory syndrome coronavirus 2 (SARS-CoV-2) tested on reverse transcription-polymerase chain reaction (RT-PCR) and 50 members of healthcare staff also tested positive. The most common diagnosis of patients was found to be stress-related adjustment disorder [16/74 (21.6%) in 2019 and 16/52 (30.8%) in 2020]. The paper discusses the common stressors voiced by the patients and their caregivers during the COVID-19 pandemic. Several challenges of providing psychological services were overcome by the team and the paper touches upon the common strategies that were used during the pandemic. Most patients did not need medications, but a significant minority did benefit from treatment with psychotropic medications. Simple psychological interventions such as sleep hygiene, supportive therapy sessions and psycho-education benefited many patients and were feasible even during the pandemic.

**Conclusion:**

The provision of psycho-oncology services to cancer patients and their caregivers was important before and during the COVID-19 pandemic.

Watch a video which illustrates the psycho-oncology service provisions in an oncology centre in Eastern India during the COVID-19 pandemic here: https://ecancer.org/en/video/9707-psycho-oncology-service-provisions-for-hospitalised-cancer-patients-before-and-during-the-covid19-pandemic.

## Background

Stress-related conditions, anxiety and depression are common in cancer patients [1]. The various risk factors for psychiatric morbidity in patients with cancer include the chronicity of the disease, relapse of cancer, premorbid psychiatric diagnosis, personality traits and economic hardships [2]. Patients with cancer benefit from integrated psychosocial care that is timely and sensitive to their needs. A recent review on the social impact of the coronavirus disease (COVID-19) pandemic on cancer patients [3] highlights some of the sources of psychological stress for cancer patients and their caregivers. In this review, the authors describe that the perception of patients ‘being at a higher risk of serious complications if infected by COVID-19’ was a source of psychological stress for patients [3]. Cancer patients experienced guilt when family members helped them with activities of daily living, often not following the guidelines of maintaining social distancing [4]. Services also faced special challenges while managing end-of-life care. Issues related to unresolved grief, non-availability of family members around the time of death, failure to say goodbye and difficulties in conducting rituals after death added to the complexities [5]. One review suggested that the COVID-19 pandemic may have posed as a barrier to cancer patients in accessing palliative care and supportive care services [6]. There is relatively less data on the psychiatric needs of hospitalised cancer patients during the COVID-19 pandemic. The present paper looks at the psychological service provision to patients admitted in a comprehensive cancer centre over two periods: once before COVID-19 and once during the COVID-19 pandemic in a cancer centre in eastern India. The provision of a timely service that addresses the mental health needs of patients is important in oncology. The present paper looks at this aspect of care amidst the COVID-19 pandemic. A nationwide surveillance study from the UK, on neurological and psychiatric complications of COVID-19, pointed out that an altered mental state was more prevalent in older patients with COVID-19 often unmasking latent neurocognitive deficits that had remained unidentified [7]. Cancer is an illness that is common in the elderly age group, it remains important that psychiatric and psychological services are available to hospitalised cancer patients during the pandemic.

## Methods

### Setting

The study was conducted at the Tata Medical Centre, Kolkata, which is a specialist comprehensive cancer centre in eastern India. The centre treats patients with cancer from eastern India, Bangladesh, Nepal, Bhutan and other neighbouring countries. The total bed capacity of the institution increased from 183 in early 2019 to 437 by early 2020 with the addition of 254 beds in phase two of the hospital in the same campus in a phased manner. The institution has had a Department of Palliative Care and Psycho-oncology since the year of inception. The psycho-oncology staff consists of two consultant psychiatrists and two fellows in psycho-oncology, both of whom are qualified clinical psychologists. In the hospital where this study was conducted, all hospitalised cancer patients needing psychiatric referrals are referred using the electronic health records system. Patients are mostly seen on the same day of referral by one of the consultant psychiatrists accompanied by a clinical psychologist. During the episode of admission of an individual patient, every patient who is referred to psycho-oncology is followed-up several times during their stay in the hospital, and a follow-up plan is made to address the psychological needs of the patient post-discharge. During this study, the retrospective data were collected from the electronic patients’ records. The study received an ethical waiver (EC/WV/TMC/54/20) from the institutional ethical review board of Tata Medical Centre, Kolkata. Inpatient psycho-oncology referral and response patterns were compared for the same 1-month period between 15th July and 15th August for 2020 (at the peak of the COVID-19 pandemic) with 2019 (as a baseline comparator). This was particularly carried out to compare the service provision before and during the COVID-19 pandemic.

### Data collection

All patients who were included in the study had been referred to the psycho-oncology department. Data were collected using a predetermined proforma. The data collected included the following: (1) referral-related data (date and time of referral, the reason for referral, referring team and admitting team), (2) demographic details of the patient (age and gender), (3) disease-related data (cancer diagnosis, medical comorbidities and reason for admission), (4) psychiatric assessment and treatment-related data (psychiatric diagnosis, nature of mental health interventions provided, details of psychotropic medications if prescribed and details of psychological interventions carried out).

### Analysis

Simple univariate statistical analysis was conducted with percentages for the patients managed during the two time periods. The medications prescribed were compared against the dosage recommended by the British National Formulary (dosage) [8] and the Maudsley Prescribing Guidelines in Psychiatry [9].

## Results

**Figure 1.**
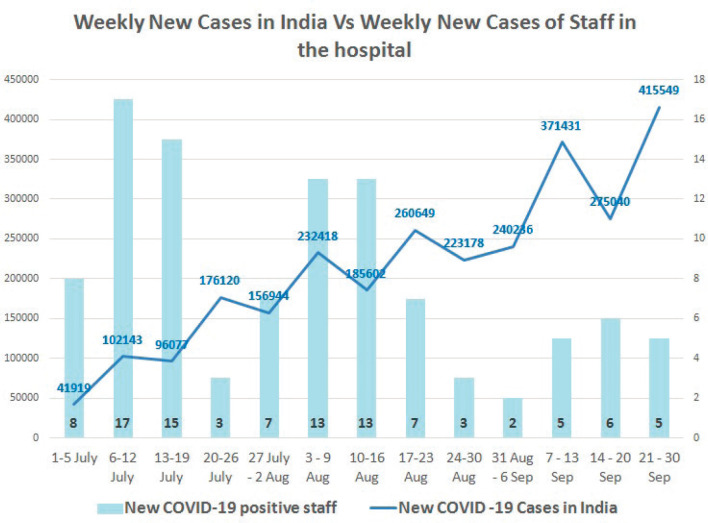
Trend of newly diagnosed cases of COVID-19 in the country and also among the hospital staff where the study was conducted.

The study was conducted amidst the COVID-19 pandemic and during this study period the number of people infected with severe acute respiratory syndrome coronavirus 2 (SARS-CoV-2) in the Indian subcontinent was on the rise. During the study period of 1 month in the year 2020, 50 members of the staff among 2,350 total staff strength in the hospital had tested positive for SARS-CoV-2 infections (Figure 1). The psycho-oncology team was involved in providing psychological support to staff as well. This experience of addressing mental health challenges of cancer care workers has been published elsewhere [10]. In the 1-month study period, 292 patients with cancer tested positive for SARS-CoV-2 in the hospital.

### Psychological services for cancer patients before and after the COVID-19 pandemic

The service offered by psycho-oncology had been in place since the year of inception of the hospital. The data from 2019 showed a representative period in the year when there was no pandemic (Table 1). The number of hospitalised cancer patients who were managed by the psycho-oncology services was 74 in 2019 and 52 in 2020 (during the peak of the COVID-19 pandemic in India). The median age of patients managed in 2019 and 2020 was 51.5 (Inter-quartile range (IQR) = 38.5–62) years and 55 (IQR = 46.25–64.25) years, respectively, but the difference was not significant statistically (*p* = 0.18, Mann–Whitney U 1,655). The median duration of hospitalisation of cancer patients who were managed by psycho-oncology services in 2019 and 2020 was 10 days (IQR = 7–14.25) and 12 days (IQR = 7–22), respectively, and the difference was not significant (*p* = 0.15, Mann–Whitney U 1,636).

Overall, the pattern suggests that patients with head and neck cancers (HNC) constituted the highest number of inpatient referrals both during and before the pandemic. HNCs are one of the most common cancers in Indian men. The referrals were made in the immediate post-operative period by the surgical oncology team looking after HNC patients. The typical HNC patient reviewed by the psycho-oncology services was a middle-aged man in the post-operative period with symptoms of anxiety that were accentuated by his inability to express his needs verbally, due to an indwelling tracheostomy tube. The second common referral in 2019 was from clinical haematology, mostly in the context of health-related anxieties for a long duration of treatment. Occasionally, the surgical and medical oncology teams wanted help from the consultant psychiatrists to manage restlessness in patients presenting with neuropsychiatric symptoms secondary to delirium, while the admitting team corrected the underlying cause of the delirium. Several patients in 2020 had tested positive for COVID-19 (7/52, 13.5%) either before or during the study period and some had an indeterminate COVID-19 test status (8/52, 15.4%) that eventually was found to be negative on repeat testing. The common reasons for referral mentioned by the referring team (Table 2) included symptoms of anxiety [7/74 (9.5%) in 2019 and 10/52 (19.2%) in 2020], low mood [14/74 (19%) in 2019 and 10/52 (19.2%) in 2020], restlessness in the context of delirium [14/74 (19%) in 2019 and 10/52 (19.2%) in 2020], problems in sleep induction and maintenance [6/74 (8.1%) in 2019 and 4/52 (7.7%) in 2020], help in assessing a child undergoing treatment for cancer [3/74 (4.1%) in 2019 and none in 2020] and opinion on the management of pre-existing psychiatric disorders needing treatment [3/74 (4.1%) in 2019 and 5/52 (9.6%) in 2020] as mentioned in the referral notes. Following psychiatric assessment, the pre-existing psychiatric conditions were found in 8.1% (6/74) of the patients in 2019 and 9.6% (5/52) of the patients referred and seen in the study period of 2020. The premorbid diagnoses in 2019 were mood disorder (*n* = 2), anxiety disorders (*n* = 2), dementia (*n* = 1) and hyperkinetic disorder (*n* = 1). In 2020, the premorbid psychiatric diagnoses made were mood disorder (*n* = 2) and anxiety disorder (*n* = 3). All referrals were seen by the psycho-oncology team on the same day of referral in 2019 and in the following year, amidst the SARS-CoV-2 pandemic, 84.6% of the referred patients were seen on the same day and 96.2% within 2 days of referral. The psychiatric diagnosis was made by consultant psychiatrists as per the International Statistical Classification of Diseases and Related Health Problems - 10th Revision (ICD-10) criteria [11] after a face-to-face interview with the patient and obtaining corroborative information from a relative or a caregiver. Following the psychiatric assessment, in both the years, a significant number of cancer patients were diagnosed to have a stress-related adjustment disorder [16/74 (21.6%) in 2019 and 16/52 (30.8%) in 2020]. In the specific month included in the audit, two patients (2/52, 3.8%) presented with suicidal thoughts in 2020 as opposed to none seen in the same month in 2019. The common stressors as enumerated by many of the patients and their family members are described in Table 3. Anxiety disorders were diagnosed in 10/74 (13.5%) patients in 2019 and a major depressive episode while being admitted in the hospital was diagnosed in 5/52 (9.6%) patients in 2020 by the psycho-oncology team during admission. It is also noteworthy that many patients reviewed by the psycho-oncology clinicians in the study periods in both the years [24/74 (32.4.%) in 2019 and 22/52 (42.3%) in 2020] did not qualify for a psychiatric diagnosis (especially those who were screened in the post-operative period or had very transient problems) and coped well during their admission in a cancer hospital for treatment. Treatment offered by the psycho-oncology services (Table 4) mostly included psychological support to patients and family members [15/74 (20.3%) in 2019 and 12/52 (23.1%) in 2020] and at times judiciously combining this with medications for a short period of time [23/74 (31.1%) in 2019 and 27/52 (32.7%) in 2020]. It is interesting to note that even in situations where psychotropic medications were prescribed, the dose of these medications never needed to be above the recommended therapeutic dosage range as suggested by the British National Formulary. In fact, several patients who were prescribed medications for short-term symptoms responded to doses lower than those used in the non-cancer patient population (Table 4).

The four types of medications that were used for psychiatric comorbidities for patients with cancer included anti-depressant medications (escitalopram, sertraline and mirtazapine) used for anxiety and depressive disorders, low-dose antipsychotic medications (aripiprazole, olanzapine and risperidone) mostly used for a short duration to manage restlessness in the context of delirium and occasional short-term use of zolpidem and benzodiazepines (clonazepam or alprazolam) when patients had isolated sleep problems during hospitalisation. The underlying cause of the delirium was managed by the admitting team and common causes of delirium were infections and electrolyte disturbances in the cancer patients. For the patients who had been diagnosed with an anxiety or depressive disorder, the medications were continued beyond admission along with psychological interventions. For those patients with restlessness, in the context of delirium, antipsychotic medications were started in a low dose and response was monitored on a daily basis by the consultant psychiatrists and the dosage required were often those below the therapeutic dose equivalents as indicated for psychosis. The anti-psychotic medications were stopped after a few days once the patient stopped being restless. Benzodiazepines were also stopped in most patents.

### Impact of the pandemic on relatives/caregivers of patients

Tata Medical Centre, Kolkata, always admits patients along with a family member or caregiver of the patient. This helps in post-discharge care planning and the relatives are encouraged to develop some of the skills needed to care for the patient when he or she is at home. This learning process is supervised by the nursing staff in the ward where the patient is admitted. This practice demystifies cancer care and fosters a relationship of trust between the family caregivers and the oncology services. Due to the constant presence of the family caregivers in the hospital while the patient remains admitted, the family members speak to the psycho-oncology team who support the family caregivers and also the patients during their hospital stay. The COVID-19 pandemic had impacted the psychological health of the relatives and the psycho-oncology team provided psychological support to them as well. The psychological challenges faced by the caregivers and relatives (described in Table 3) could be broadly divided into three categories: (a)* ‘Fear of contracting COVID-19 due to their presence in a hospital which was perceived to be a high-risk environment’*; (b)* ‘Stress related to commuting from home to hospital to access care in the initial period of lockdown’*; (c)* ‘Stress related to the financial impact of COVID-19 due to the broader economic impact of the pandemic resulting in loss of earnings’*. During the specific 1-month audit period in 2020, 4/52 (7.6%) of the relatives of index cancer patients received formal psychiatric/psychological interventions independently. These four patients had either a mood disorder or a stress-related condition. One of them was a couple who received formal couple’s therapy from a mental health professional.

**Figure 2.**
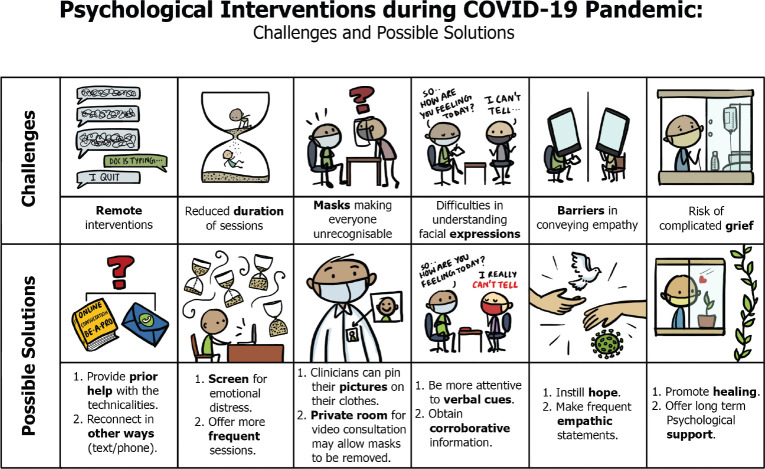
Challenges and possible solutions for providing psychological interventions to cancer patients during the COVID-19 pandemic.

Providing psychological services during the COVID-19 pandemic was challenging due to several reasons and these are described in brief in Figure 2 along with the ways in which the team members adapted their functioning to circumvent these challenges.

The various psychological techniques that helped patients were providing psychological supportive therapy to the patient [14/74 (18.9%) in 2019 and 10/52 (19.2%) in 2020], allowing patients to express their worries [8/74 (10.8%) in 2019 and 9/52 (17.3%) in 2020], psychoeducation [9/74 (12.2%) in 2019 and 3/52 (5.8%) in 2020], discussing sleep hygiene techniques [4/74 (5.4%) in 2019 and 5/52 (9.6%) in 2020], providing psychological support to family member/caregiver [5/74 (6.8%) in 2019 and 2/52 (3.8%) in 2020] and facilitating environmental modifications in patients with delirium [13/74 (17.6%) in 2019 and 4/52 (7.7%) in 2020]. Most patients were seen several times during the period of admission by the psycho-oncology clinicians. The psychological interventions were individualised according to the needs of the patient and were delivered in the mother tongue of the patient by the multi-lingual group of psycho-oncology clinicians all of whom spoke multiple Indian languages.

### Institutional impact of the SARS-CoV-2 pandemic

**Figure 3.**
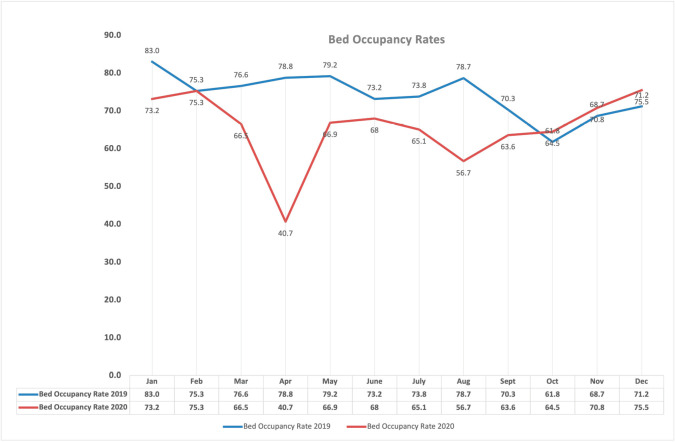
Comparative bed occupancy rates of the institution.

**Figure 4.**
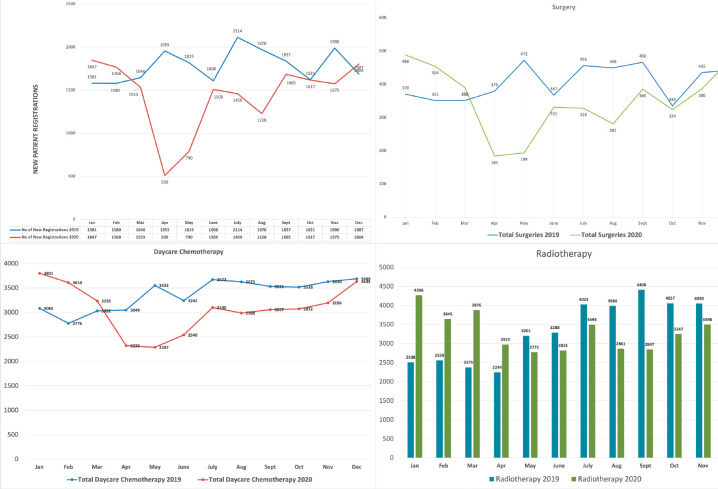
Comparative clinical activity in 2019 and 2020.

The institution continued to provide cancer care for the patients without any interruptions during the pandemic with lesser number of patients attending the hospital specifically at two time points: at the initial lockdown period starting in March–April 2020 and also in August–September 2020 when the pandemic reached its peak in India. This pattern can be easily identified in Figure 3 that shows the comparative bed occupancy rates prior to the pandemic and during the pandemic in 2020. Figure 4 shows the comparative clinical activity in terms of new patient registrations, number of surgeries performed, day care chemotherapies undertaken and radiotherapies delivered.

The overall footfall in the hospital, the numbers of surgeries performed, the number of chemotherapies administered as a day care procedures and radiotherapies undertaken all followed the same pattern (Figure 4).

The institution was prompt to develop a command centre to coordinate the clinical activities so that there is minimum disruption of care. There were COVID-19-specific measures taken that are described in the next section.

### Post-COVID-19 management of patients

A consolidated clinical protocol on management of cancer patients during the COVID-19 pandemic was formulated with multidisciplinary inputs from various specialists incorporating the national and international guidelines. The hospital identified specific locations for the management of COVID-19 suspects and COVID-19-positive cancer patients. Two beds were earmarked in the emergency room and respiratory isolation ward, with separate entry and exit, personal protective equipment (PPE) donning and doffing areas and dedicated patient pathways for holding COVID-19 suspects, collection of samples and management of respiratory distress. There was a negative pressure isolation ward established in the new phase two of the hospital that had seven beds for admitting and treating COVID-19-positive patients. Later on during the pandemic, a separate intensive care unit (ICU) with negative pressure ventilation provisions was earmarked as ‘COVID ICU’ having a capacity of nine beds. Psycho-oncology referrals could be made from any of these areas. The commonest reason for referral for cancer patients admitted during active COVID-19 infection was due to restlessness associated with delirium. In the post-COVID-19 phase after the patients were discharged home, the common psychiatric diagnosis was depression and anxiety when patients were reviewed in the outpatient clinics. The consultant in infectious diseases managed these outpatient clinics. Psycho-oncology services were involved in assessing and managing the psychological aspect of these patients recovering from COVID-19 after these patients were referred by the infectious disease specialists.

### Impact of the COVID-19 pandemic on staff

**Figure 5.**
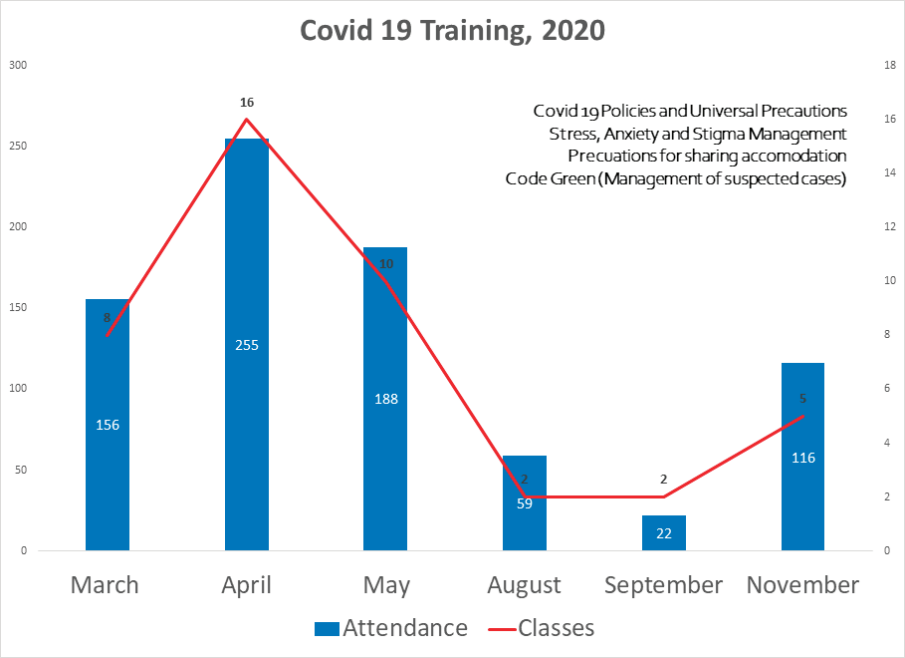
Training sessions for staff during the pandemic.

As there was a significant degree of stress among staff, from the beginning of the pandemic several training sessions were conducted for various staff groups. The training sessions were conducted virtually or face-to-face maintaining social distancing in an open air space of the hospital to reduce the chances of infection. The topics that were discussed included ‘donning and doffing of gloves with demonstration’, ‘stigma reduction for being a COVID-19 warrior’, ‘managing suspected COVID-19-positive patients’, ‘stress/anxiety management’, ‘precautions around staying in shared accommodation’ and other topics that were relevant to the staff group attending the training. Figure 5 shows the total number of training sessions conducted from March to November 2020 and also the monthly attendance of staff for these sessions. Some of the training was conducted for line managers and they cascaded the information to respective staff groups. The sessions were often conducted jointly by a consultant psychiatrist and a public health specialist and often joined by a consultant microbiologist and infectious disease specialist. Staff were offered accommodation nearby the hospital during the lockdown period (due to difficulties in commuting) and also if they became COVID-19-positive (if they wanted to stay away from home for fear of passing on the infection to others). Food and refreshments were provided free of charge. Human resources department and finance department ensured that staff were paid a few days in advance than their usual salary date, so that they felt reassured about the institution being supportive of them.

## Discussion

The present study is a description of the provision of psycho-oncology services in a multidisciplinary cancer centre in India prior to and during the COVID-19 pandemic. Cancer care was complemented by psychological and psychiatric care of patients and caregivers who were referred to psycho-oncology services both prior to and during the COVID-19 pandemic. The majority of referred patients, who qualified for a psychiatric condition, had a diagnosis of stress-related psychiatric condition as an adjustment disorder, anxiety disorder or depression. Previous research has shown that depression and anxiety often coexist at a syndrome [12] and symptom level [13] in the population. This was the case in our study population as well.

Interventions consisted of either only psychological interventions or a combination of medications and psychological interventions or medications only. However, most patients required short-term, less-complex interventions, except in those situations where the patient had a premorbid psychiatric condition or in rare circumstances of serious psychiatric morbidity at the time of cancer treatment.

One of the limitations of the present study is having a relatively small sample size that was not powered to detect the differences in the diagnostic trends statistically between the two study periods. We have thus represented the data in simple percentage terms. However, given the fact that the study was conducted at a time when the SARS-CoV-2 pandemic was on the rise in India and very few cancer centres managed to provide routine psycho-social care to patients with cancer, we believe the findings of this study will be of interest to the readers. The other limitation worth discussing is that the study included patients who were referred to the psycho-oncology services based in the same hospital and these figures should not be used to calculate the frequency of certain psychiatric diagnoses in any specific subgroup of cancer patients as universal screening of all patients was not feasible. The strength of the study was that it compared the service provisions to internationally accepted diagnostic and treatment standards. The study represented the day-to-day psycho-oncology service provision following a pragmatic design without any specific exclusion criteria. The psychological assessments of hospitalised cancer patients covered in this paper had been conducted face-to-face with appropriate PPE during the time of the pandemic.

Previous publications on prescribing patterns of psychotropic medications for patients with unipolar depression and stress-related conditions showed that around 10% of these patients may not be prescribed any antidepressant medications [14]. However, in an oncology set-up, the frequency of prescribing psychotropic medications is often less. Our study showed that in oncology a large number of patients may be adequately managed by psychoeducation, discussing sleep hygiene techniques and other supportive psychological management strategies. When medications were used, our study showed that we never needed high doses of psychotropic medications and, in fact, many patients achieved symptom control in doses lower than those used in non-oncology patient populations. Our findings are in keeping with the results reported by a systematic review of prescribing practices of antidepressants in cancer patients showing a prevalence rate of prescription of antidepressant medications being 15.6%, with even lower prescription rates for studies originating in Asia included in the same review [15].

Presently, there is a movement towards person-centred psychiatric care and individualising the care based on the needs of the patient being given the priority [16]. This is in keeping with our approach to providing psycho-oncological care to patients with cancer. COVID-19 pandemic posed several challenges to organising healthcare services. As discussed elsewhere in the world [17], team working and solidarity with patients, their family members and our colleagues likely went a long way to help us overcome the challenges we faced in providing mental health services amidst the COVID-19 pandemic. We hope the lessons learned during the pandemic will provide unique insights into future service delivery models in other low- and middle-income countries.

## Conclusion

Hospitalised cancer patients can have a co-morbid psychiatric diagnosis and benefit from these problems being formally assessed by mental health professionals. Psycho-oncology services when integrated with mainstream oncology services can respond quickly to the needs of patients with cancer. The COVID-19 pandemic has posed several challenges in addressing the mental health needs of patients with cancer, but the present study showed that it is possible to continue these services even amidst the pandemic. Further research is needed on the organisation of psycho-oncology services so that optimal and timely response to the mental health needs of cancer patients can be planned.

## Conflicts of interest

The authors declare no conflict of interest.

## Authors’ contributions

Study conception and design: Soumitra S. Datta and Arnab Mukherjee; Data collection: Meheli Chatterjee, Shreshta Chattopadhyay, Chitralekha Bhowmick, Archisman Basu, Surya Bhattacharjee and Soumita Ghose; Write-up: Soumitra S. Datta, Arnab Mukherjee and Soumita Ghose; Approval of final manuscript: All authors.

## Figures and Tables

**Table 1.** Characteristics of hospitalised cancer patients who were referred to psycho-oncology services during a period of 1 month in pre-COVID pandemic times (in 2019) and during the peak of the COVID-19 pandemic (in 2020).

Variable		2019		2020	
		(*n* = 74)	(%)	(*n* = 52)	(%)
Age (in years)		51.5 (IQR 38.5 – 62)		55 (IQR 46.25 – 64.25)	
Gender	Female	29	39.2	20	38.5
	Male	45	60.8	32	61.5
Domicile	West Bengal	51	68.9	41	78.8
	Bihar	4	5.4	3	5.8
	Jharkhand	5	6.8	2	3.8
	Orissa	2	2.7	1	1.9
	Other Indian states	3	4.1	5	9.6
	Bangladesh	9	12.2	0	0
Educational level attained	No formal education	10	13.5	14	26.9
	Primary school	13	17.8	7	13.5
	High school	9	12.2	2	3.8
	Graduation	24	32.4	16	30.8
	Post-graduation	13	17.6	3	5.8
	Other professional qualifications	5	6.7	10	19.2
Occupation	Unemployed	23	31.08	24	46.2
	Government employee	4	5.4	5	9.6
	Private company employee	5	6.7	5	9.6
	Businessman	11	14.9	7	13.5
	Professional (doctor, lawyer etc)	7	9.4	2	3.8
	Semi-skilled worker	5	6.8	2	3.8
	Homemaker	9	12.2	5	3.8
	Student	10	13.5	2	3.8
Duration of hospitalisation (in days)		10 (IQR 7–14.25)		12 (IQR 7–22)	
Cancer type	HNC	27	36.5	28	53.8
	Breast cancer	3	4.1	2	3.8
	Lung cancer	5	6.8	3	5.8
	GI cancers	11	14.9	6	11.5
	Gynaecological cancers	7	9.5	6	11.5
	Urological cancers	1	1.4	3	5.8
	Haematological cancers	18	24.3	4	7.7
	Other cancers	2	2.7	0	0
Co-morbidities (one person may have more than one co-morbidities)	Hypertension	23	31.1	22	42.3
	Diabetes	15	20.3	15	28.8
	Hypothyroidism	8	10.8	8	15.4
	Others	20	27	19	36.5
COVID status	Positive	NA		7	13.5
	Indeterminate	NA		8	15.4
	Negative	NA		37	71.2
Admitting team	Surgical Oncology	33	44.6	35	67.3
	Medical Oncology	9	12.2	6	11.5
	Clinical Haematology	12	16.2	3	5.8
	Others	20	27	8	15.8
Reason for admission	Surgery	31	41.9	34	65.4
	Chemotherapy	9	12.1	3	5.7
	Management of co-morbidities (including infections)	22	29.7	5	9.6
	GI symptoms (including intestinal obstructions)	4	5.4	4	7.7
	Symptom management (including pain)	6	8.1	5	9.6
	Pre-habilitation	1	1.35	1	1.9
	Organic brain syndrome	1	1.35	0	0

**Table 2.** Psychiatric referral, response of psycho-oncology services and psychiatric diagnosis of hospitalised cancer patients who were referred to psycho-oncology services during a period of 1 month in pre-COVID pandemic times (in 2019) and during the peak of the COVID-19 pandemic (in 2020).

Variable		2019		2020	
		**(*n* = 74)**	**(%)**	**(*n* = 52)**	**(%)**
Reasons for referral	Child and family assessment of paediatric cancer patients	3	4.1	0	0
	Screening for emotional distress in post-operative period	24	32.4	20	38.5
	Sleep problems	6	8.1	4	7.7
	Symptoms of anxiety	7	9.5	10	19.2
	Low mood	14	19	10	19.2
	Pre-existing psychiatric disorder and on treatment	3	4.1	5	9.6
	Restlessness secondary to delirium	9	12.2	1	1.9
	Pre-habilitation	1	1.4	1	1.9
	Organic brain syndrome	1	1.4	0	0
	Not mentioned	6	8.1	1	1.9
					
Assessment by consultant psychiatrist within	Same day	74	100	44	84.6
	within 1 day			3	5.8
	within 2 days			3	5.8
	Within 1 week			2	3.8
					
Psychiatric diagnosis	Delirium	13	17.6	5	9.6
	Adjustment disorder	16	21.6	16	30.8
	Depressive episode	2	2.7	5	9.6
	Anxiety disorders	10	13.5	1	1.9
	Substance related problems	4	5.4	1	1.9
	Organic mood disorder	4	5.4	0	
	Schizophrenia	0	0	1	1.9
	Other psychiatric disorder	1	1.4	1	1.9
	No psychiatric diagnosis	24	32.4	22	42.3

**Table 3.** Anecdotal sources of stresses reported by patients and family members during the COVID-19 pandemic.

	Source of stress for cancer patients	Source of stress for family/caregivers of cancer patients
Social construct of COVID-19	1. Some cancer patients were worried about contracting COVID-19 infection.	1. Some family members were scared of supporting the patient due to fear of contracting COVID-19.
	2. If they contracted COVID-19 infection, during isolation, they were often reminded of their illness (cancer) and the usual sources of support were cut-off temporarily from them which was stressful.	
Barriers to access oncology service	1. Majority of the patients wanted to continue their cancer treatment.	1. Family members found it difficult to commute to cancer centres due to the lock down in the early days.
	2. If for some reasons there were interruptions in their oncology treatment, this was a source of worry for them.	
Treatment seeking and treatment adherence	1. Several factors led to delay in treatment seeking for some of the patients who were newly diagnosed with cancer outside the hospital.	1. Family members found it difficult to commute and care of the patients
Economic issues	1. Economic hardships were felt by many patients.	1. Overall economic hardships were felt by family members and caregivers as well. This is particularly important in many low and middle income countries (LMICs) where oncology treatment is often funded by out-of-pocket expenses of patients and his or her family members.
	2. Several patients were apprehensive of losing their job as they viewed cancer to be a major disadvantage in the milieu of the broader economic downturn.	

**Table 4.** Psychological treatment details and psychotropic medication used for hospitalised cancer patients who were referred to psycho-oncology services during a period of 1 month in pre-COVID-19 pandemic times (in 2019) and during the peak of the COVID-19 pandemic (in 2020).

Variable		2019		2020	
		(*n* = 74)	(%)	(*n* = 52)	(%)
Treatment given	Psychological treatment only	15	20.3	12	23.1
	Medication only	16	21.6	4	7.7
	Both psychological and medication treatment	23	31.1	17	32.7
	None needed	20	27	19	36.5
Type of psychological intervention	No psychological intervention	21	28.4	19	36.5
	Psychoeducation	9	12.2	3	5.8
	Allowing to express worries/ventilation	8	10.8	9	17.3
	Sleep hygiene	4	5.4	5	9.6
	Providing support to the family	5	6.8	2	3.8
	Supportive therapy to patient	14	18.9	10	19.2
	Environmental modification	13	17.6	4	7.7
Number of psychotropic medications prescribed	None	37	50	31	59.6
	One	30	40.5	17	32.7
	Two	7	9.5	4	7.7
Classification of daily dosage according to British National Formulary (BNF) [Same patient may be on more than one medication]	Therapeutic range	20	48.8	12	48
	Responded below therapeutic range	21	51.2	13	52
	Needed medication above therapeutic range	0	0	0	0
Medications prescribed (Same patient may have been prescribed more than one medication)	Escitalopram	1	2.4	1	4
	Sertraline	3	7.3	0	0
	Mirtazapine	3	7.3	4	16
	Aripiprazole	11	26.8	2	8
	Olanzapine	7	17.1	1	4
	Risperidone	6	14.6	3	12
	Lorazepam	3	7.3	2	8
	Clonazepam	5	12.2	11	44
	Alprazolam	1	2.4	1	4
	Zolpidem	1	2.4	0	0

## References

[1] Couper JW, Pollard AC, Clifton DA (2013). Depression and cancer. Med J Aust.

[2] Anuk D, Özkan M, Kizir A (2019). The characteristics and risk factors for common psychiatric disorders in patients with cancer seeking help for mental health. BMC Psychiatry.

[3] Tsamakis K, Gavriatopoulou M, Schizas D (2020). Oncology during the COVID-19 pandemic: challenges, dilemmas and the psychosocial impact on cancer patients. Oncol Lett.

[4] Sirintrapun SJ, Lopez AM (2018). Telemedicine in cancer care. Am Soc Clin Oncol Educ Book.

[5] Morris SE, Paterson N, Mendu ML (2020). Grieving and hospital-based bereavement care during the COVID-19 pandemic. J Hosp Med.

[6] Al-Quteimat OM, Amer AM (2020). The impact of the COVID-19 pandemic on cancer patients. Am J Clin Oncol.

[7] Varatharaj A, Thomas N, Ellul MA (2020). Neurological and neuropsychiatric complications of COVID-19 in 153 patients: a UK-wide surveillance study. Lancet Psychiatry.

[8] Joint Formulary Committee British National Formulary [Internet]. https://www.bnf.org].

[9] Taylor DM, Barnes TRE, Young AH (2018). The Maudsley Prescribing Guidelines in Psychiatry.

[10] Datta SS, Mukherjee A, Ghose S (2020). Addressing the mental health challenges of cancer care workers in LMICs during the time of the COVID-19 pandemic. JCO Glob Oncol.

[11] World Health Organization (2004). ICD-10: International Statistical Classification of Diseases and Related Health Problems.

[12] Kalin NH (2020). The critical relationship between anxiety and depression. Am J Psychiatry.

[13] Fava M, Alpert JE, Carmin CN (2004). Clinical correlates and symptom patterns of anxious depression among patients with major depressive disorder in STAR*D. Psychol Med.

[14] Grover S, Kumar V, Avasthi A (2012). An audit of first prescription of new patients attending a psychiatry walk-in-clinic in north India. Indian J Pharmacol.

[15] Sanjida S, Janda M, Kissane D (2016). A systematic review and meta-analysis of prescribing practices of antidepressants in cancer patients. Psychooncology.

[16] Boardman J, Dave S (2020). Person-centred care and psychiatry: some key perspectives. BJPsych Int.

[17] Kelly BD (2020). Coronavirus disease: challenges for psychiatry. Br J Psychiatry.

